# Behavioral Disorders of Spatial Cognition in Patients with Mild Cognitive Impairment due to Alzheimer’s Disease: Preliminary Findings from the BDSC-MCI Project

**DOI:** 10.3390/jcm13041178

**Published:** 2024-02-19

**Authors:** Davide Maria Cammisuli, Valeria Isella, Federico Verde, Vincenzo Silani, Nicola Ticozzi, Simone Pomati, Virginia Bellocchio, Valentina Granese, Benedetta Vignati, Gloria Marchesi, Lorenzo Augusto Prete, Giada Pavanello, Gianluca Castelnuovo

**Affiliations:** 1 Department of Psychology, Catholic University, 20123 Milan, Italy; davide.cammisuli1@unicatt.it; 2 Department of Neurology, School of Medicine, University of Milano-Bicocca, 20126 Milan, Italy; valeria.isella@unimib.it; 3 Milan Center for Neurosciences, 20133 Milan, Italy; 4 Department of Neurology and Laboratory of Neuroscience, IRCCS Istituto Auxologico Italiano, 20149 Milan, Italy; f.verde@auxologico.it (F.V.); vincenzo@silani.com (V.S.); n.ticozzi@auxologico.it (N.T.); 5 Dino Ferrari Centre, Department of Pathophysiology and Transplantation, University of Milan, 20122 Milan, Italy; 6 Neurology Unit, Luigi Sacco University Hospital, 20157 Milan, Italy; simone.pomati@asst-fbf-sacco.it; 7 Catholic University, 20123 Milan, Italy; virginiabellocchio12@gmail.com (V.B.); valentina.granese@unicatt.it (V.G.); benedetta.vignati01@icatt.it (B.V.); gloria.marchesi03@icatt.it (G.M.); 8 School of Specialization in Clinical Psychology, Catholic University, 20123 Milan, Italy; lorenzoaugusto.prete@unicatt.it (L.A.P.); giada.pavanello01@icatt.it (G.P.); 9 Clinical Psychology Research Laboratory, IRCCS Istituto Auxologico Italiano, 20149 Milan, Italy

**Keywords:** spatial disorientation, mild cognitive impairment, Alzheimer’s disease, wearable technology, hippocampus

## Abstract

(1) Background: Spatial cognition (SC) is one of the earliest cognitive domains to be impaired in the course of Alzheimer’s disease (AD), resulting in spatial disorientation and becoming lost even in familiar surroundings as later dementia symptoms. To date, few studies have identified initial alterations of spatial navigation (SN) in the premorbid AD phase by real-world paradigms, and none have adopted an innovative technological apparatus to better detect gait alterations as well as physiological aspects correlated to spatial disorientation (SD). The present study aimed at exploring initial SN defects in patients with prodromal AD via a naturalistic task by using a sensory garment. (2) Methods: 20 community-dwelling patients with Mild Cognitive Impairment (MCI) due to AD and 20 age/education controls were assessed on their sequential egocentric and allocentric navigation abilities by using a modified version of the Detour Navigation Test (DNT-mv). (3) Results: When compared to controls, patients with MCI due to AD exhibited higher wrong turns (WT) and moments of hesitation (MsH) in the DNT-mv, reflecting difficulties both in sequential egocentric and allocentric navigation, depending on hippocampal deterioration. Moreover, they reported more complaints about their SN competencies and lower long-term visuospatial memory abilities than controls. Remarkably, WTs and MsH manifested in the allocentric naturalistic task of the DNT-mv were associated with autonomic nervous system alteration pertaining to cardiac functioning in the whole sample. (4) Conclusions: Naturalistic navigation tests of hippocampal function using a continuous non-invasive monitoring device can provide early markers of spatial disorientation in patients with MCI due to AD. Future studies should develop cognitive remediation techniques able to enhance SC residual abilities in patients at high risk of conversion into dementia and ecological paradigms to be replicated on a large scale.

## 1. Introduction

Alzheimer’s disease (AD) is characterized by progressive deterioration of cognitive functions with episodic memory loss and spatial disorientation as main hallmarks [1]. Spatial cognition is one of the earliest cognitive domains to be impaired in patients with AD, resulting in spatial disorientation (SD) [1]. Advances in biomarkers and behavioral tests for identifying individuals at higher risk of developing AD are a crucial target of current research. No causal therapies for AD are currently available, and considering SD as a cognitive biomarker of AD, interventions that may help in early preserving SN abilities may contribute to counteract dementia progression [2].

Spatial navigation (SN) is supported by multiple parallel representations, including egocentric (body-centered) navigation, allocentric (world-related), and pure path integration (PI) [3,4]. As strong evidence of the sectorial literature, egocentric frames define spatial positions using the navigator’s body as a point of reference, while allocentric reference frame codes the position of the target relative to surrounding landmarks and their reciprocal spatial relationship, independently from the observer’s position. Such information is thus used to build up a cognitive map of the environment, an internal representation of the spatial scenario [3]. Path integration is also particularly important because it permits one to estimate distances and navigate environments without using landmarks [4], allowing an individual to perform an online self-localization during the navigation. Iglói and colleagues [5] specifically defined ‘sequential egocentric navigation’ as the ability to recall multiple body turns associated with choice points that are learned by their order along a path, thus sharing a similarity with episodic memory.

The sequential egocentric strategy differs from path integration in the sense that it does not permit an online localization of the navigator, but it allows the acquisition of spatial sequences associated with the activation of the left hippocampus, that is, the recounting of left-right body turns sequence to retrace a route [6]. People adopt mixed strategies for real-life SN, with the dorsal striatum and posterior parietal cortex considered the key structure of simple-response egocentric navigation, the hippocampus providing allocentric environmental representations, and retrosplenial cortex and parietal–occipital sulcus allowing both types of representations to interact [3,4]. Neuropathological studies have also suggested that early AD pathology affects the entorhinal cortex (EC), harboring grid cells, a brain area associated with path integration (PI) [7].

Medial temporal lobe (MTL) structures, including the hippocampus, are critical for episodic memory, too. Particularly, the hippocampus plays an integral role in the episodic memory process [8] and binding of multimodal information [9]. In an animal model, Rondi-Reid and colleagues also found that mice lacking N-methyl-D-aspartate (NMDA) receptors in the CA1 of the hippocampus showed a deficit in sequential egocentric strategy, suggesting a role of the CA1 in the acquisition of these specific memory traces [6]. Consistent findings for studies in humans have shown decreased memory performances, such as consolidation into long-term storage associated with NMDA receptor hypofunction [10].

Spatial disorientation has been reported in most AD patients, and it is considered one of the first symptoms of the disease [1]. This behavioral feature causes patients to make SN errors in the community and subsequently can result in becoming lost or wandering even in familiar environments during the advanced phases of the disease [11,12], contributing to augmenting caregivers’ burden. Spatial disorientation has been reported in AD and MCI patients as deficits in free recall or temporal order of the landmarks, route learning, landmarks recognition and location on a 2D map, route drawing, and evaluating directions [12].

Recently, SD revealed in real-world navigation tasks was suggested to be particularly sensitive to detect the earliest cognitive signs of AD [13] and to predict the development of pre-dementia syndromes [14]. There is also evidence to suggest that SN abilities decline in the course of physiological aging [15]. The severity of SN decline in aging was shown to be apparent, especially in route learning tasks of naturalistic experiments that become progressively prominent as age increases [16].

Spatial navigation skills are compromised early in MCI, regardless of its subtype (i.e., single or multiple domains) [17]. Moreover, amyloid-positive MCI patients perform worse than amyloid-negative MCI patients in egocentric and allocentric navigation tasks use fewer shortcuts, move more slowly, and stay longer at crossings in a realistic spatial task of searching for objects’ location [17].

It has been suggested that early gait alterations can be used as biomarkers of cognitive impairment since they are present in dementia [18] and even related to the disease stages [19]. Finally, heart rate variability (HRV), reflecting the balance between acceleratory sympathetic and inhibitory parasympathetic systems, is mediated by specific brain areas involved both in cognitive processing and in autonomic regulation of cardiovascular function and may vary during stressful experiences [20]. In line with this, it deserves to be studied in-depth during SN tasks.

To date, no naturalistic investigations have explored behavioral disturbances of spatial cognition in senior citizens with MCI due to AD by using an effective technological apparatus able to support experimental observations about gait and physiological correlates that may be associated with altered performances. Thus, the present study aimed to fill this literature gap by using a continuous non-invasive monitoring device in patients with prodromal AD able to record physiological and gait parameters during a cutting-edge naturalistic SN task.

## 2. Materials and Methods

### 2.1. Participants

A total of 20 community-dwelling patients with MCI due to AD (M = 15; F = 5), as well as 20 healthy controls (M = 8; F: 12), were recruited for the study. All patients were clinically diagnosed with MCI using Petersen’s diagnostic criteria [21], and prodromal AD was defined according to Dubois and colleagues’ criteria [22,23]. For all participants, the inclusion criteria encompassed the following ones: age between 65 and 85 years; education not less than 5 years; no dementia (a Montreal Cognitive Assessment score > 18.28, according to Santangelo et al. [24]); basic ICT skills. The exclusion criteria were having a previous history of alcohol/substance abuse, diagnosis of neurological/psychiatric condition, or any other significant medical conditions that significantly interfere with SN (e.g., head injury, vision loss, mobility issues).

### 2.2. Technological Apparatus: Sensory Smart Textile and Digital Health Applications

Before the navigation of an urban garden, all participants were equipped with the Howdy Senior System© (Comftech S.r.l., Monza, Lombardy, Italy). The system consists of the Howdy Senior electronic unit (a class IIB medical device), the ‘Howdy Senior App’ for patient’s vital data monitoring on the clinician’s smartphone, and a sensory garment (a class I medical device) to be worn by participants during the experimental task. Among vital parameters recorded by sensors and reported on the Howdy Senior App, we specifically collected some cardiac and respiratory functions. Within cardiac functions, we collected time-domain over RR intervals parameters, such as the stress index [24] and the LF/HF (LF = power in the low-frequency band affected by Sympathetic Nervous System; HF = power in the high-frequency band affected by Parasympathetic Nervous System) ratio. Within respiratory functions, we collected the avgBR (mean of breath rate) and the SDBR (standard deviations of breath rate) parameters. The Howdy Senior App is also able to collect the following accelerometer parameters: AccXavg, AccYavg, and AccZavg (i.e., mean of accelerations for X, Y, and Z axes); AccXsd, AccYsd, and AccZsd (i.e., the standard deviation of accelerations for X, Y, and Z axes); and AccXrms, AccYrms, and AccZrms (i.e., root mean square of accelerations for X, Y, and Z axes).

The Howdy Senior System has been recently implemented by a GPS mobile application for gait analysis investigation, determining the GPX tracks of body position and movements around urban environments. Such tracks were elaborated by another app, the ‘Aptive’ one. Beyond ‘Latitude’, ‘Longitude’, and ‘Altitude’, the Aptive App is able to record the following variables: ‘Time’ as the number of minutes to complete a predefined route; ‘Speed’ as walking velocity provided by geolocation; ‘Direction’ corresponding to gait orientation with values ranging from 0° to 360°, where 0° corresponds to the North, 90° to the East, 180° to the South and 270° to the West.

Physiological parameters were collected for all participants on a 5 min rest state prior to the naturalistic experiment and during the four phases of the DNT-mv (i.e., *Route A* outward; *Route A* return; *Route B* outward; and *Route B* return). Gait data were collected for all participants during the four phases of the DNT-mv.

### 2.3. Real-Space Navigation Testing

**Figure 1:**
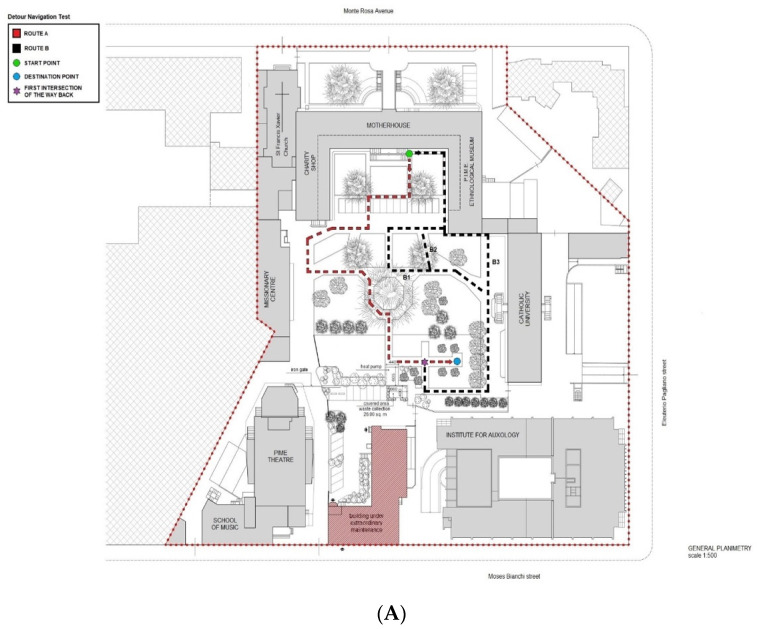

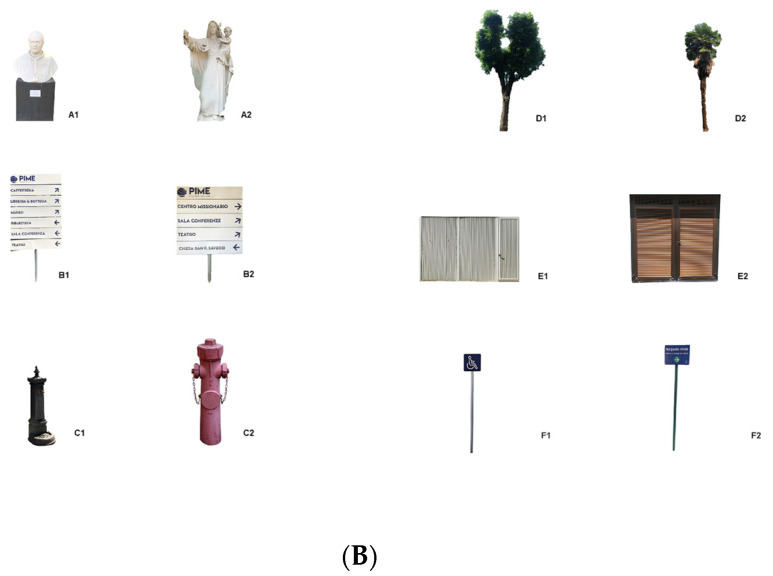
(**A**). The DNT-mv was performed in the outdoor garden of the Pontificio Istituto Missioni Estere—PIME (81, Monterosa Street, Milan, Italy), a 1.000 m^2^ arena (20 × 50 m^2^). General planimetry on a scale of 1:500. (**B**) PIME landmarks. Hits on the left column; distractors on the right column.

All participants underwent a modified version of the Detour Navigation Test (The DNT-mv). Differently from the original paradigm [25], we set up an ecological task exploring SN alterations in patients with prodromal AD when traveling to an unfamiliar location since they are aware of their neighborhoods and they do not become lost during routine community walks, as happens in patients with advanced dementia. The whole urban park circuit is represented in Figure 1A. The participants are asked to wear the sensory garment prior to the experiment at the hospital. Then, they were accompanied to the urban park adjoining the hospital. The urban park is surrounded by buildings, therefore not adjacent to urban roads and closed to motor vehicle traffic. This location was chosen to ensure the safety of the participants during the experiment and to avoid confounding variables.

The participants were first asked to complete a path from a start point (i.e., the green point, Figure 1A) to a destination point (i.e., the blue point, Figure 1A) of the *Route A* (i.e., the *Route A* outward) via an investigator-guide walk (Figure 1A, dotted red line; 80.5 m, on a scale of 1 to 500). The participant is also asked to pay attention to all landmarks that he/she will find along the way and informed that at a certain point along the route (i.e., about half of the route), he/she will be asked to perform a simple algebraic operation (i.e., subtracting 7 from 100 and progressing further) without stop walking till the destination point. We envisaged this interfering task to mimic the distracting stimuli of an urban route (e.g., car horns, background noise, a familiar person who greets, a personal phone call while walking, etc.), making the experiment fully ecological.

Once they arrived at the destination point, they were asked whether the pictures shown by the investigator via a booklet were the correct landmarks (n.6) they had encountered during the encoding phase or whether they were distractors (n.6) similar to the landmarks, for a total of 12 pictures (Figure 1B).

After the landmarks recognition task, the participants were required to navigate back to the start point using the same route alone (i.e., the *Route A* return). Feedback was provided by the investigator in case of deviation from the original path, in order to provide a retrieval cue of the correct direction. Such a kind of spatial cognition task entails participants predominantly using an egocentric sequential navigation based on self-centered information, a visual processing of a consecutive sequence of turn-points in relation to the body movements. Then, the participants were required to reach the destination point of the encoded route again (i.e., the *Route B* outward). However, at the first intersection of the way back (i.e., Figure 1A, purple asterisk), unknown to them, they were asked to find an alternative route back, i.e., the *Route B* return (*Route B*^1^ return, 94 m; *Route B*^2^ return, 78.5 m; and *Route B*^3^ return, 74 m. Figure 1A, dotted black lines) that does not overlap with the *Route A* at all. This kind of spatial cognition task mainly requires participants to utilize an allocentric strategy based on a formed cognitive map of the urban garden. For the *Route B* return, the investigator followed the examinees without giving any feedback. The whole DNT-mv took about 20 min to be administered.

Both for *Route A* and *Route B* return, we detected two route disorientation scores, i.e., ‘wrong turns’ (WTs) and ‘moments of hesitation’ (MsH), as already described in Puthusseryppady and colleagues [25]. Accordingly, all the violations in terms of deviations from the pre-established path (i.e., movement at an intersection onto a different path as compared to the original one or non-roadable walkways) were written down by the experimenter as WTs in a pre-set paper grid for the *Route A* return while the overlaps with the *Route A* return, and the non-roadable walkways were written down by the experimenter as WTs in a pre-set paper grid for the *Route B* return. The sum of mistakes of the *Route A* and of the *Route B* return constituted the WTs score.

Further, we detected an MH as the participant either slows down/stops and looks around to aid orientation or verbally admits to the experimenter that he/she is unsure about his/her whereabouts, both for the *Route A* and the *Route B* return. We calculated the time spent for each hesitation by a stopwatch. The sum of hesitation times constituted the MsH score in seconds. In addition to the experimenter’s observation and annotation on the pre-set paper grid, we measured the MsH more objectively by using the Howdy Senior integrated accelerometer results. To this end, we used the motion sensor of the wearable garment, measuring the individual’s linear acceleration in the three axes (i.e., x, y, z). To segment the magnitude signals, according to Ghosh et al. [26], we adopted time intervals of 10 s with an overlap of 5 s between subsequent intervals because a 10 s segment is sufficiently long to capture multiple steps during walking, thus revealing important behavioral changes. In this way, we detected each MH as a signal flattened along the three axes.

At the end of the DNT-mv, the participants were returned to the hospital and administered the Corsi learning suvra-span (CLSS) [27], the Subjective Spatial Navigation Complaints (SSNC) questionnaire [28], and the Geriatric Depression Scale (GDS) [29] in order to evaluate long-term visuospatial memory, self-perception of navigational guidance skills, and mood, respectively. The SSNC questionnaire was back-translated for Italian language and cultural adaptation.

### 2.4. Statistical Analysis

We performed a controlled experiment. The normality of the collected data was tested by means of the Shapiro–Wilk. Independent samples *t*-tests were used for normally distributed data and Mann–Whitney *U*-tests were used when data did not distribute normally. A non-parametric analysis (i.e., Bonferroni-corrected Mann–Whitney *U*-test) was then applied to compare groups’ performances on WTs and MsH of the *Route A* and *Route B* return of the DNT-mv, physiological parameters (stress index, LF/HF, agvBR, and SDBR), and gait data (Aptive Time, Speed, and Direction) as well as on the Corsi learning suvra-span, SSNC, and GDS. A *p*-value < 0.05 was set to reach significance. Further, Spearman’s rank correlations on the whole sample and on subgroups were also used to further investigate the association among the neuropsychological (CLSS) and clinical measures (SSNC and GDS) measures, the route disorientation scores (WTs and MsH both for *Route A* and *Route B* return) and the Aptive variables (Time, Speed, and Direction).

All analyses were run by the Statistical Package for the Social Sciences (SPSS) software for Windows (SPSS, version 23.0; SPSS, Inc., Chicago, IL, USA). We also used the MATLAB toolbox 2023b (The MathWorks Inc., Natick, MA, USA) to translate the Aptive App signals into binary and CSV files prior to the SPSS elaboration.

## 3. Results

### 3.1. Demographics

Participants’ demographics are shown in Table 1. When comparing patients and controls on demographics variables, the groups were matched according to age (t = 0.393, *p* = n.s.), education (U = 142,500, *p* = n.s.), but not for gender (χ^2^ = 5013; *p* = 0.025) and global cognition (MoCA) (U = 94.500, *p* = 0.007).

### 3.2. Naturalistic Test Findings and Related Measures

The patients with MCI due to AD significantly presented with more WTs and MsH than controls on Route A return (WTs, U = 51.500, *p* = 0.001, η^2^ = 0.346; MsH, U = 65.000, *p* = 0.001, η^2^ = 0.290) and Route B return (WTs, U = 83.000, *p* = 0.009, η^2^ = 0.146; MsH, U = 79.000, *p* = 0.004, η^2^ = 0.229). The groups did not differ in terms of correct landmarks recognition (i.e., hits) (U = 51.500, *p* = n.s.) or choice (alternative paths) of Route B return (χ^2^ = 3702; *p* = n.s.) whereas patients with MCI due to AD showed lower performance on the CLSS (U = 36.000, *p* = 0.004, η^2^ = 0.245) and on the SSNC questionnaire (U = 28.000, *p* = 0.006, η^2^ = 0.218) than counterparts. No difference regarding GDS score was found between groups (*p* = n.s.) (Table 2).

### 3.3. Physiological Parameters and Gait Data Collected via the Digital Apps

Physiological parameters collected for all participants on the rest state prior to the naturalistic experiment and during the four phases of the DNT-mv (*Route A*, outward; *Route A*, return; *Route B,* outward; *Route B,* return) were shown in Table 3. Gait data collected for all participants during the four phases of the DNT-mv are shown in Table 4. The groups did not show any significant difference in physiological and gait parameters (*p* = n.s.).

### 3.4. Correlations

**Figure 2:**
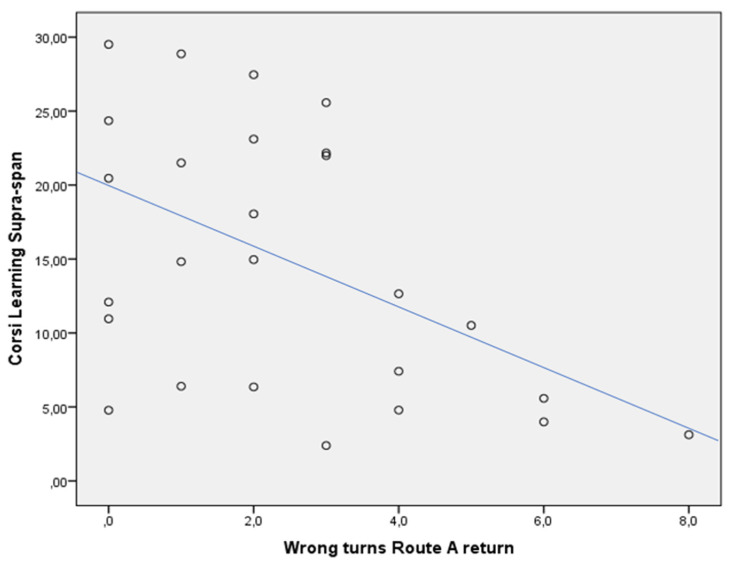
Dispersion graph illustrating the correlation between the Corsi Learning suvra-span (CLSS) and wrong turns (WT) of the Route A return in the whole sample. X axis = number of wrong turns of the Route A; Y axis = Corsi Learning suvra-span score.

**Figure 3:**
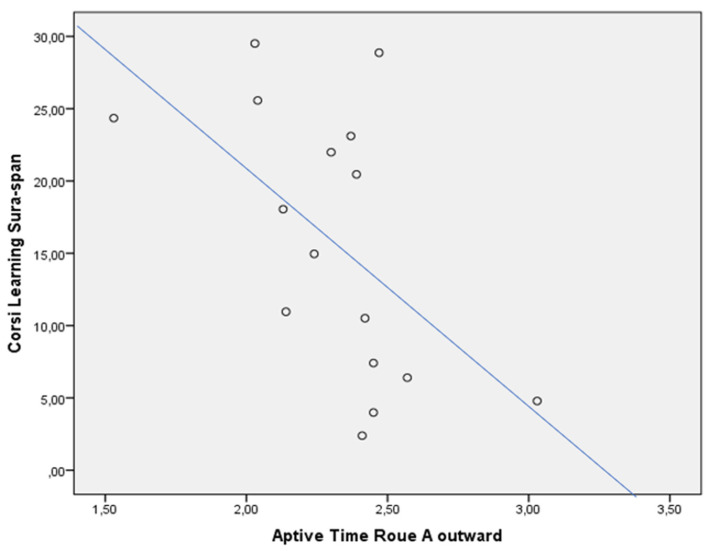
Dispersion graph illustrating the correlation between the Corsi Learning suvra-span (CLSS) and Aptive Time Route A outward in the whole sample. X axis = time in minutes to complete the Route A, outward; Y axis = Corsi Learning suvra-span score.

**Figure 4:**
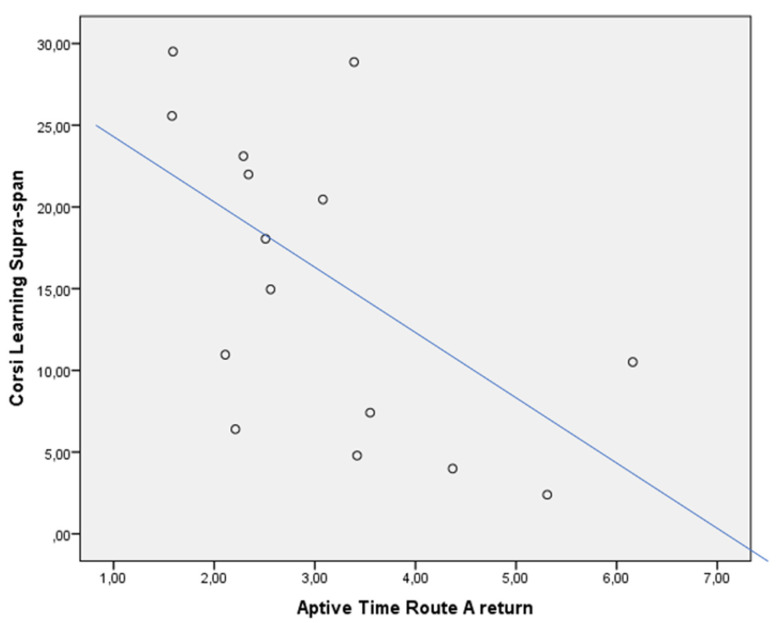
Dispersion graph illustrating the correlation between the Corsi Learning suvra-span (CLSS) and Aptive Time Route A return in the whole sample. X axis = time in minutes to complete Route A, return; Y axis = Corsi Learning suvra-span score.

In the whole sample, the CLSS was negatively associated with WTs of the Route A return (rho = −0.425, *p* < 0.05) (Figure 2), and Aptive Time Route A outward (rho = −0.605, *p* < 0.05) (Figure 3) and return (rho = −0.621, *p* < 0.05) (Figure 4). This was not confirmed in the subgroups, both for patients with MCI due to AD (CLSS vs. Route A WTs: rho = −0.405, *p* > 0.05; CLSS vs. Aptive Time Route A outward: rho = −0.319, *p* > 0.05; CLSS vs. Aptive Time Route A return: rho = −0.371, *p* > 0.05) and controls (CLSS vs. Route A WTs: rho = 0.058, *p* > 0.05; CLSS vs. Aptive Time Route A outward: rho = −0.200, *p* > 0.05; CLSS vs. Aptive Time Route A return: rho = −0.143, *p* > 0.05).

**Figure 5:**
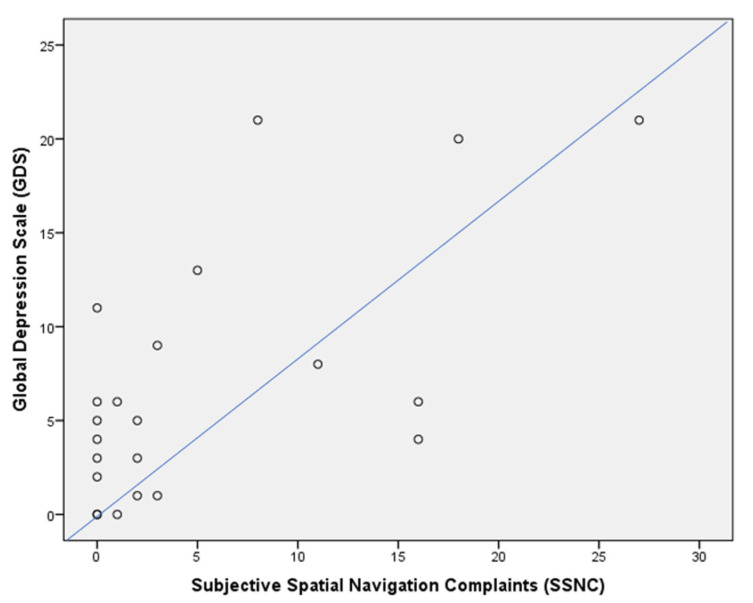
Dispersion graph illustrating the correlation between the Subjective Spatial Navigation Complaint (SSNC) questionnaire and the Global Depression Scale (GDS) in the whole sample. X axis = Subjective Spatial Navigation Complaints score; Y axis = Geriatric Depression Scale score.

For the entire sample, the SSNC was positively associated with the GDS (rho = 0.530, *p* < 0.01) (Figure 5). This was not confirmed in the subgroups, both for patients with MCI due to AD (rho = 0.436, *p* > 0.05) and controls (rho = 0.436, *p* > 0.05).

**Figure 6:**
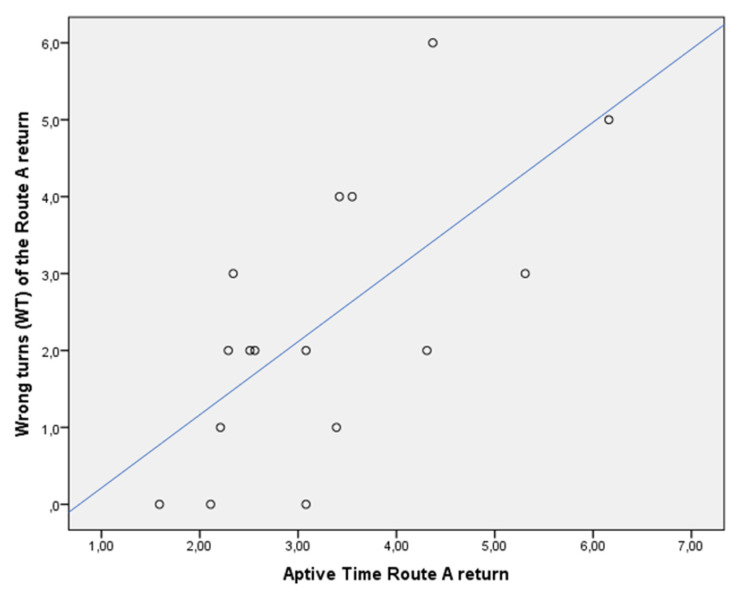
Dispersion graph illustrating the correlation between wrong turns (WT) of the Route A return and Aptive Time in the whole sample. X axis = time in minutes to complete the Route A, return; Y axis = number of wrong turns of the Route A, return.

**Figure 7:**
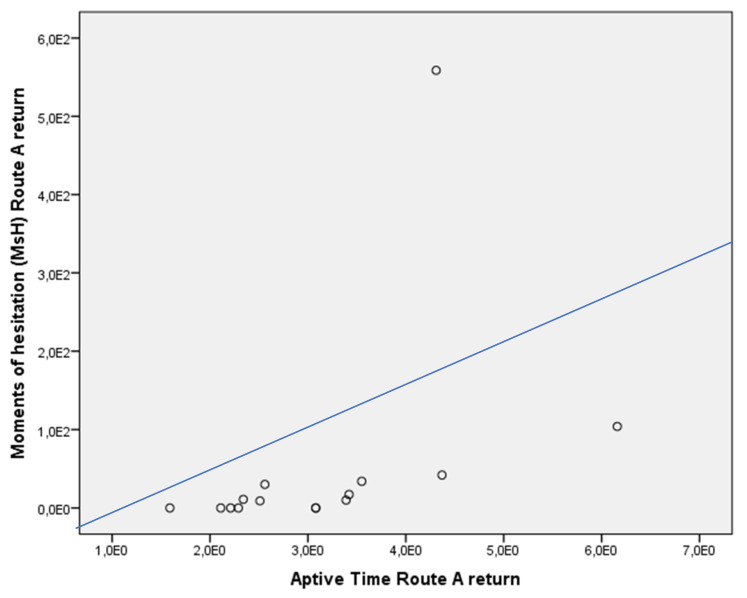
Dispersion graph illustrating the correlation between moments of hesitation (MsH) of the Route A return and Aptive Time in the whole sample. X axis = time in minutes to complete the Route A, return; Y axis = moments of hesitation in seconds during the Route A, return. Variables were expressed in scientific notation.

Both WTs and MsH of the Route A return were positively associated with Aptive Time (rho = 0.833, and rho = 0.810, *p* < 0.01, respectively) in the whole sample (Figure 6 and Figure 7). This was not confirmed in the subgroups, both for patients with MCI due to AD (WTs Route A return vs. Aptive time: rho = 0.327, *p* > 0.05; MsH Route A return vs. Aptive Time: rho = 0.750, *p* > 0.05) and controls (WTs Route A return vs. Aptive time: rho = 0.243, *p* > 0.05; MsH Route A return vs. Aptive Time: rho = 0.412, *p* > 0.05).

**Figure 8:**
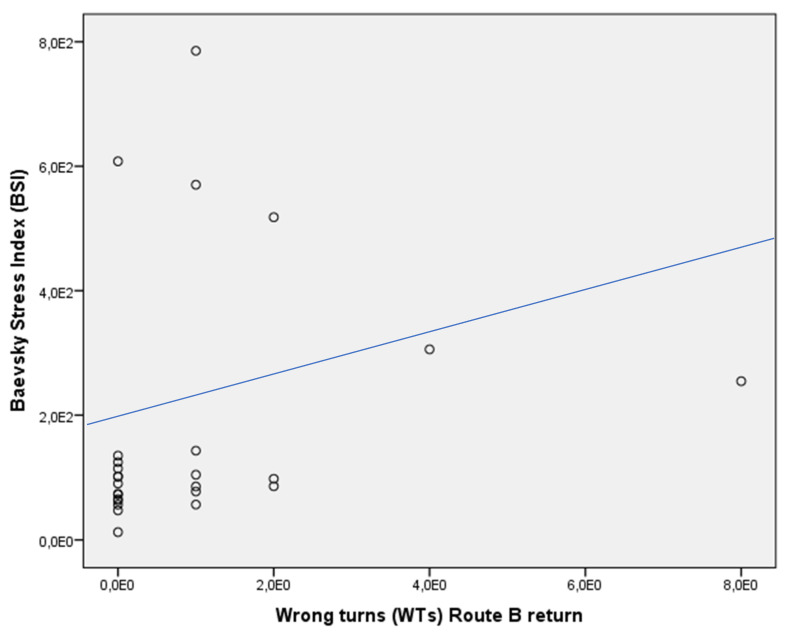
Dispersion graph illustrating the correlation between Baevsky stress index (BSI) and wrong turns (WTs) of the Route B return in the whole sample. X axis = number of wrong turns of the Route B, return; Y axis = Baevsky stress index output of the formula. Variables were expressed in scientific notation.

In the whole sample, the Baevsky stress index positively correlated with WTs of the Route B return (rho = 0.407, *p* < 0.05) (Figure 8). This was not revealed in the subgroups of patients with MCI due to AD (Baevsky stress index vs. WTs Route B return: rho = 0076, *p* > 0.05) as well as controls (Baevsky stress index vs. WTs Route B return: rho = 0.076, *p* > 0.05).

No other correlation among the variables under consideration was found in the whole sample and when subgroups were compared.

## 4. Discussion

The novelty of this study is based on the fact that we first explored SN deficits by using a modified ecological paradigm of route learning and the formation of a spatial map of mental representation in patients with prodromal AD using an innovative technological apparatus able to collect physiological and gait parameters during the experiment. Our study confirms that Mild Cognitive Impairment due to AD is associated with behavioral alterations when navigating in real-world settings, such as an enclosed circular arena [30,31] and hospital surroundings [32,33]. We specifically documented such alterations in terms of WTs and MsH both for *Route A* and *Route B* return, reflecting difficulties in sequential egocentric strategy and allocentric navigation, respectively. Poor performances in hippocampal-dependent navigation tasks have been reported as a specific characteristic of age-related cognitive decline [16]. However, we documented that SN alterations we found as WTs and MsH were more pronounced in patients with AD biomarkers than in healthy controls, depending on hippocampal deterioration.

With regard to WTs, spatial features such as environmental complexity and the number of turns along the path to be learned affect spatial and temporal memory efficiency [34]. In particular, hippocampal activity is associated with the encoding of turns positions into long-term memory [34]. According to Schapiro et al. [35], turns along a route also allow humans to extract regularities from the environment and segment temporally extended experiences. Both for the *Route A* and *Route B* return, we verified that such a kind of navigation modality deteriorated in MCI patients with AD biomarkers, causing them to make numerous wrong turns in the route-retracing task and the difficulty to finding an alternative route without making mistakes along the way (i.e., overlaps with the *Route A* return, or non-roadable walkways crossing) to reach the start point of the naturalistic task, respectively. This observation is consistent with the fact that the hippocampus is one of the earliest brain regions affected by AD, and spatial memory tasks of hippocampal function have the potential to detect MCI due to AD [36].

With regard to MsH, by analyzing differences between groups and processing accelerometric data collected, patients with MCI due to AD exhibited hesitant walking both in *Route A* and *Route B* return because more variation has been observed in their step intervals when compared to controls that, in turn, were more confident during walking, reporting further uniform step intervals. According to Schaat et al. [37], these data are able to carefully analyze the person’s walking modality and represent the most informative locomotion features of spatially disoriented behavior. By this means of analysis, we bypassed methodology restraints reported by Puthusseryppady and colleagues in their investigation [25], too. Findings pertaining to MsH underlined the poor hippocampal efficiency of MCI patients with AD biomarkers once again. Particularly, Nedelska and colleagues [38] reported smaller right hippocampal volume in patients with amnestic MCI and mild-to-moderate AD when compared to controls, as brain alteration associated with poor allocentric navigation evaluated in a real-space version of the human Morris water maze task.

In typical AD, initial pathological changes in MTL structures are particularly associated with long-term visuospatial memory. Patients with MCI due to AD significantly had a failure in CLSS when compared to controls, as expected. However, it has been shown that visuospatial memory is heavily dependent on brain structures, which exhibit a particular vulnerability both to normal aging and degenerative dementia to a different extent [39]. This can explain the correlation between the CLSS and the WTs of the *Route A* return. As widely recognized, episodic memory decline occurs in the elderly while it specifically defines AD in its early stage as reduced encoding and rapid forgetting of new material [40]. The poor efficiency of the long-term visuospatial memory revealed by performance on CLSS would determine the necessity to use more attentional resources to learn a new path and to replicate it backward, traveling down the path more slowly. This can explain the correlation between the CLSS and the Aptive Time of the *Route A* outward and return.

The patients with MCI due to AD also reported more complaints of self-perceived spatial navigation decline in familiar and unfamiliar surroundings as well as its direct impact on daily functioning than controls, as already reported in the literature [28]. We also documented that the higher this self-perception, the more depressed the mood, confirming that depression in old age is associated with greater complaints about one’s cognitive status, including SN skills [41].

Changes in step length and time spent to complete a path characterize outdoor behavior in the elderly [42]. Research has shown that older adults experience an alteration of gait when cognitive tasks are performed during walking, a phenomenon referred to as dual-task interference [42]. The interfering task we entered in our naturalistic experiment (i.e., the algebraic calculation) weighed equally on the two groups that took longer to successfully complete the *Route A* return, resulting in a higher amount of time spent in mistakes made along the route (WTs) and in hesitation (MsH). We did not find a significant difference in recognition of landmarks between patients and controls at the end of the DNT-mv learning phase (i.e., the *Route A* outward). According to a previous investigation [43], landmark recognition may engage different brain regions than landmark mapping, including more posterior medial temporo-occipital areas that may severely deteriorate in more advanced stages of AD as atrophy spreads into these regions.

We also found that the route disorientation scores for the *Route B* return were associated with heart rate variability (HVR) parameters. Particularly, difficulties in reproducing the correct turns (WTs) in wayfinding were associated with the stress index [24], an indicator of the sympathetic regulation preparing the organism for fight-flight reactions [44].

Our study presents some limitations. First, our findings are based on real-world SN experiments, which may be very difficult to implement broadly. Second, another group of comparison consisting of individuals with subjective cognitive decline (SCD) should be recruited to further explore potential differences along the clinical course of AD since subjective navigation complaints have been reported in this population, too [28]. Furthermore, learning and recalling spatial information of a new route is affected in MCI, and the deficit severity may be located between the performance of healthy older adults and individuals with SCD [45]. Third, virtual navigation measures obtained by the Starmaze Task [46], mainly evaluating sequential egocentric navigation, and the Sea Hero Quest (SHQ, www.seaheroquest.com, accessed on 10 January, 2024) [47] mainly evaluating allocentric navigation, respectively, should be used for complete assessment as well as structural MRI. Finally, it would be of interest the investigation of spatial anxiety, to ascertain the influence of worry that some behavioral tasks in real-world settings might induce in older adults [40].

According to Kirova et al. [48], MCI is a critical period during which cognitive restructuring and neuroplasticity occur. Therefore, cognitive remediation therapies of spatial cognition could have a beneficial effect on decreasing the likelihood of a more severe cognitive deterioration during the AD course. To this end, we suggest that computer-based cognitive exercises enhancing long-term visuospatial memory and immersive virtual reality training, such as the NeuroVirtual 3D [49], should be adopted as complementary parts of a training protocol for the rehabilitation of SN deficits in MCI due to AD, with the final aim of improving SC. Finally, recent guidelines indicate the importance of promoting multidimensional interventions to prevent further decline in MCI, emphasizing the use of technology [50]. Particularly, telerehabilitation systems have the potential to engage patients in programs encompassing cognitive and motor activation by guaranteeing a low-cost continuum of personalized care via remote control by therapists [51].

## 5. Conclusions

The pathophysiology of AD is associated with MTL dysfunction. Hippocampal atrophy specifically predicts MCI conversion into AD, and its deterioration is associated with SN impairment in everyday activities, such as difficulties in finding one’s way in an unfamiliar environment for patients in the prodromal phase [46].

We demonstrated that real-world navigation testing supported by a specialized technical apparatus recording physiological and gait parameters represents a promising approach to identifying SN deficits in MCI patients with AD biomarkers early. Despite the ecological value of our findings, future research should be oriented toward designing more replicable real-life paradigms and in arranging rehabilitation protocols capable of enhancing residual SN abilities in MCI patients with AD biomarkers to counteract the progression of the neurodegenerative process.

## Figures and Tables

**Table 1:** Participants’ demographics. MCI due to AD = Mild Cognitive Impairment due to Alzheimer’s disease; HCs = healthy controls; MoCA = Montreal Cognitive Assessment. Variables were expressed as mean ± SD for age (in years), median value for education (in years) and global cognition (test score), and percentage for gender.

Demographic Variables	MCI due to AD (*n*. 20)	HCs (*n*. 20)	Significance
Age (yrs.)	70.98 ± 4.75	72.26 ± 5.99	*p =* n.s.
Education (yrs.)	9.50	13.00	*p =* n.s.
Gender	M = 75%; F = 15%	M = 40%; F = 60%	*p = 0.025*
MoCA score	21.79	24.52	*p = 0.015*

**Table 2:** The Detour Navigation Test and related measures results. WTs = wrong turns (number of, n.); MsH = moments of hesitation (seconds, s); CLSS = Corsi learning suvra-span; SSNC = Subjective Spatial Navigation Complaints; GDS = Global Depression Scale; MCI due to AD = Mild Cognitive Impairment due to Alzheimer’s disease; HCs = healthy controls. Variables were expressed as median values.

DMT-mv and Related Measures	MCI due to AD (*n*. 20)	HCs (*n*. 20)	Significance
Route A return, WTs (n.)	4	1	*p* = 0.001
Route A return, MsH (s)	44	0	*p* = 0.001
Route B return, WTs (n.)	1	0	*p* = 0.009
Route B return, MsH (s)	14	0	*p* = 0.004
CLSS score	5.99	21.98	*p* = 0.004
SSNC score	8	1	*p* = 0.006
GDS score	3.5	7.5	*p* = n.s.
Route A outward, landmarks recognition score	14.4	2	*p* = n.s.
Route B returns, alternative paths choice	B1 = 0; B2 = 13; B3 = 3	B1 = 3; B2 = 10; B3 = 2	*p* = n.s.
	Route A return, overlap = 4	Route A, return overlap = 5	

**Table 3:** Physiological parameters collected at rest and during the DNT-mv via the Howdy Senior digital app. MCI due to AD = Mild Cognitive Impairment due to Alzheimer’s disease; HCs = healthy controls. Variables were expressed as median values. Baevsky Stress index (BSI) = index of regulation strain characterized by the activity of sympathetic regulation. The BSI was calculated by the following equation: A*M*_o_ X 100%/2*M*_o_ X *M_x_DM_n,_* where mode (*M*_o_*)* was the most frequent NNI (RR intervals) expressed in seconds, and the amplitude of mode (A*M*_o_) was the percentage of the total measured NNI that included *M_o_*; the variation range (*M_x_DM_n_*) was the difference in seconds between the longest NNI (*M_x_*) and the shortest NNI (*M_n_*) and indicated the degree of variability in the interval; LF/HF = ratio between the power in the low- and high-frequency band (in Hz); agvBR = mean of breaths per minute (b/m); SDBR = standard deviation of agvBR.

Physiological Parameters	MCI Due to AD (*n*. 20)	HCs (*n*. 20)	Significance
Rest state			
Cardiac functions			
BSI output	9.88	6.50	*p =* n.s.
LF/HF (Hz)	2.24	2,.21	*p =* n.s.
Respiratory functions			
agvBR (b/m)	23.40	20.60	*p =* n.s.
SDBR (SD b/m)	20.36	5.84	*p =* n.s.
Route A, outward			
Cardiac functions			
BSI output	88.63	85.11	*p =* n.s.
LF/HF (Hz)	2.28	0.66	*p =* n.s.
Respiratory functions			
agvBR (b/m)	26.30	24.96	*p =* n.s.
SDBR (SD b/m)	34.44	4.98	*p =* n.s.
Route A, return			
Cardiac functions			
BSI output	87.66	93.88	*p =* n.s.
LF/HF (Hz)	2.47	0.82	*p =* n.s.
Respiratory functions			
agvBR (b/m)	28.44	26.29	*p =* n.s.
SDBR (SD b/m)	22.97	6.19	*p =* n.s.
Route B, outward			
Cardiac functions			
BSI output	105.46	109.31	*p =* n.s.
LF/HF (Hz)	3.71	1.05	*p =* n.s.
Respiratory functions			
agvBR (b/m)	27.49	26.56	*p =* n.s.
SDBR (SD b/m)	26.08	5.68	*p =* n.s.
Route B, return			
Cardiac functions			
BSI output	99.61	94.97	*p =* n.s.
LF/HF (Hz)	1.66	0.85	*p =* n.s.
Respiratory functions			
agvBR (b/m)	23.55	27.24	*p =* n.s.
SDBR (SD b/m)	33.20	5.43	*p =* n.s.

**Table 4:** Gait data collected during the DNT-mv via the Aptive digital app. MCI due to AD = Mild Cognitive Impairment due to Alzheimer’s disease; HCs = heathy controls. Time = number of minutes to complete a predefined route; Speed = walking velocity provided by geolocation expressed as meters/seconds (m/s); Direction = main gait orientation (expressed as degrees) maintaining during the ecological task, according to the cardinal points (0° = North; 90° = East; 180° = South; 270° = West). Variables were expressed as median values.

Gait Data	MCI Due to AD (*n*. 20)	HCs (*n*. 20)	Significance
Route A, outward			
Aptive Time (min)	2.45	2.19	*p =* n.s.
Aptive Speed (m/s)	0.50	0.61	*p =* n.s.
Aptive Direction (degrees)	177.90	199.39	*p =* n.s.
Route A, return			
Aptive Time (min)	3.55	2.51	*p =* n.s.
Aptive Speed (m/s)	0.43	0.60	*p =* n.s.
Aptive Direction (degrees)	149.68	142.58	*p =* n.s.
Route B, outward			
Aptive Time (min)	2.14	2.04	*p =* n.s.
Aptive Speed (m/s)	0.80	0.70	*p =* n.s.
Aptive Direction (degrees)	193.50	195.08	*p =* n.s.
Route B, return			
Aptive Time (min)	2.07	1.42	*p =* n.s.
Aptive Speed (m/s)	0.57	0.71	*p =* n.s.
Aptive Direction (degrees)	139.40	116.07	*p =* n.s.

## Data Availability

The datasets presented in this article are not readily available because they are part of an ongoing study.
